# New paradigms in research on *Dirofilaria immitis*

**DOI:** 10.1186/s13071-023-05762-9

**Published:** 2023-07-21

**Authors:** Timothy G. Geary

**Affiliations:** 1 Institute of Parasitology, McGill University, Ste-Anne-de-Bellevue, QC Canada; 2 School of Biological Sciences, Queen’s University-Belfast, Belfast, Northern Ireland

**Keywords:** Heartworm, *Dirofilaria immitis*, Vaccine, Transgenesis, Drug resistance

## Abstract

**Background:**

Since the advent of ivermectin (along with melarsomine and doxycycline), heartworm has come to be viewed as a solved problem in veterinary medicine, diminishing investment into non-clinical research on *Dirofilaria immitis*. However, heartworm infections continue to pose problems for practitioners and their patients and seem to be increasing in frequency and geographic distribution. Resistance to preventative therapies (macrocyclic lactones) complicates the picture. The use of chemotherapy for other kinds of pathogens has benefitted enormously from research into the basic biology of the pathogen and on the host-pathogen interface. A lack of basic information on heartworms as parasites and how they interact with permissive and non-permissive hosts greatly limits the ability to discover new ways to prevent and treat heartworm disease. Recent advances in technical platforms will help overcome the intrinsic barriers that hamper research on *D. immitis*, most notably, the need for experimentally infected dogs to maintain the life cycle and provide material for experiments. Impressive achievements in the development of laboratory animal models for *D. immitis* will enhance efforts to discover new drugs for prevention or treatment, to characterize new diagnostic biomarkers and to identify key parasite-derived molecules that are essential for survival in permissive hosts, providing a rational basis for vaccine discovery. A ‘genomics toolbox’ for *D. immitis* could enable unprecedented insight into the negotiations between host and parasite that enable survival in a permissive host. The more we know about the pathogen and how it manipulates its host, the better able we will be to protect companion animals far into the future.

**Graphical Abstract:**

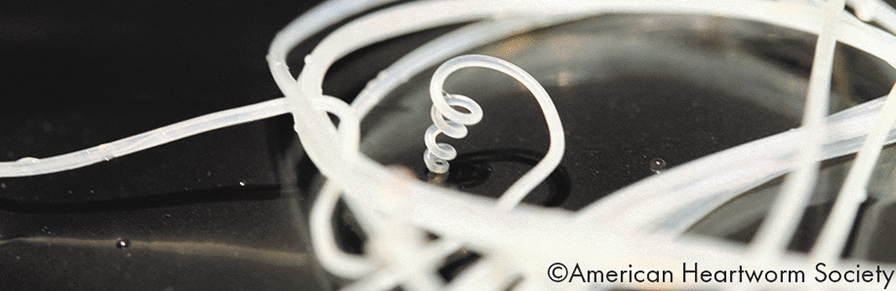

## Background

The mosquito-borne parasitic nematode *Dirofilaria immitis*, the canine heartworm, is a common pathogen in temperate and tropical parts of the Americas and other countries around the world [1]. Permissive definitive mammalian hosts in which the parasite can reproduce include most prominently canids, but also felines. Infection can lead to severe pathology and even death because of the presence of the large adult stages in the pulmonary vasculature and sometimes the heart. The degree of pathology is generally correlated with the number of adult parasites present (the worm burden); the L1 larval stages (microfilariae, mf) released from fertilized adult females are required for transmission by mosquitos but seem to cause relatively little pathology on their own [2].

Treatment of an established heartworm infection in dogs or cats is a complex and expensive process, relying on the use of an arsenical drug (melarsomine), often preceded by a month-long course of doxycycline to remove the symbiotic bacteria *Wolbachia* before killing the adults, which appears to reduce the pathology associated with the generation of large dead worms in the host [3, 4]. Prior to the discovery, development and introduction into the market in the 1980s of ivermectin (IVM), the prototype macrocyclic lactone (ML), prevention of infection was achieved with daily doses of diethylcarbamazine (DEC) during mosquito season, necessitating the routine attention of the human responsible for the animal’s care. The introduction of IVM, and subsequently a number of other MLs [3, 5] in several formulations, revolutionized heartworm prevention in companion animals by virtue of a once-monthly (and now much longer) dosing schedule; it also created a source of income and profit for veterinarians in endemic areas and animal health companies.

However, the appearance of ML-resistant strains of *D. immitis* in the USA has led to a re-evaluation of the current landscape for control of this parasite in companion animals [6]. Coupled with the realization that compliance with and adherence to heartworm prevention protocols are far from optimal, the time is ripe for reinvigorating research on this parasite. Although many aspects of heartworm research remain daunting, as noted in the next section, recent advances in other areas of research provide grounds for optimism, and these are the main focus of this article.

## Heartworm research bottlenecks

Research on medical parasitology diminished since the threat of parasitic infections in developed countries in the North and West was largely eliminated following the end of World War II and the almost complete eradication of pathogens such as malaria, *Trichinella* and hookworms due to improved sanitation, improved chemotherapeutic options and government intervention. That chemotherapy can play important roles in campaigns to control parasites, along with the economies of scale realized by intensive plant and livestock agriculture, led at the same time to increasing investments in plant protection and animal health. Prevention and control of arthropods and helminths became essential for large-scale monoculture and intensive livestock operations and resulted in the discovery of a wide range of safe, effective and inexpensive chemicals for use in veterinary medicine, some of which were then adopted for use in humans. Subsequent realization that companion animals comprised a lucrative market without the cost limitations encountered in the treatment of livestock led to the development of medicines for the prevention and treatment of ecto- and endoparasites of dogs and cats, most initially adopted from prototypes originally developed for production animals or plant protection from arthropod pests.

This era saw evolution of a burgeoning animal health industry, with multiple companies investing in the discovery and development of parasiticides for veterinary use. High safety and prolonged efficacy were achieved with excellent companion animal products for the control of fleas, ticks, mites, gastrointestinal nematodes (GIN) and heartworms. Prior to the development of resistance to these products, initially in livestock parasites, antiparasitic discovery and development became less urgent, as the parasite problem was considered solved, and consolidation of companies invested in animal health began apace. Today, very few companies are focused on de novo discovery of new antiparasitics for animal health, and the situation for human health is also limited, particularly in the major pharmaceutical companies [7, 8].

Academic research in developed countries into parasites and parasitic diseases remains an unfortunately minor component of Western/Northern veterinary schools (with some notable exceptions) despite continuing parasite problems in veterinary medicine, including perhaps most notably the development and spread of pathogens resistant to available antiparasitic drugs. Research on parasitic helminths is particularly challenging, as the absolute requirement for a host to complete the life cycle is essential for virtually all species of interest. For filarial nematodes such as *D. immitis*, the life cycle includes both an arthropod and a mammalian host, adding to the cost and complexity of maintaining parasite populations for research. This is acutely problematic for heartworms, as dogs are the preferred definitive host, and research on dogs is both very expensive and fraught with political and emotional challenges. We remain unable to establish the complete parasitic life cycle of almost all helminth pathogens in culture, and little research is apparently being conducted to overcome this severe limitation.

Modern biomedical research has developed a truly remarkable toolbox for producing recombinant animals, including mammals, arthropods and the free-living nematode *Caenorhabditis elegans*. The ‘toolbox’ for *C. elegans* [9] includes methods for efficiently and specifically altering the genome to create transgenic animals in which a particular gene is removed from or added to the genome, or in which specific mutations are made in a targeted gene, and reducing the expression of specific genes using RNA interference (RNAi). Unfortunately, these tools have rarely been adapted for routine use with medically or economically important parasitic nematodes, especially filariae such as *D. immitis*. Recent developments may help reduce this significant gap and provide reasons for optimism, as discussed below.

## New research paradigms

Advances in modern biomedical research, perhaps most dramatically in cancer biology and medicine, have revolutionized treatment options by deepening our understanding of how tumors evade and dampen normally effective immune mechanisms that eliminate altered cells before they become pathological. These advances are exemplified by the application of genome sequencing and recombinant DNA technologies on tumors and hosts to illuminate key aspects of the process of tumor development and spread. Similar, though perhaps less profound, advances have been made in understanding determinants of pathogenesis and infection with viruses, bacteria, fungi and some protozoal parasites. The integrated use of specialized animal models and genetically modified cells/pathogens has led to a much clearer understanding of the host-tumor/pathogen interface, with important therapeutic implications. Currently, achievements in these areas for parasitic nematodes, including *D. immitis*, lag far behind work on tumors and other pathogens, but there are reasons for hope. These reasons include the development of analytical tools to define the molecular dialog that characterizes the host-parasite interface, the development of laboratory animal models for important filarial parasites, including *D. immitis*, and the advent of new vaccine technologies that could foster renewed interest in heartworm vaccines. The discussion of these reasons for optimism begins with animal models.

## Development of laboratory animal models for heartworm infections

As noted, heartworms are generally quite restricted in host range (achieving reproductive success only in canids, felids and ferrets). An hypothesis to explain at least part of this phenomenon is that mammalian hosts have evolved highly effective immune responses that detect and eliminate the vast majority of parasite species before an infection can establish and that a successful infection requires that the parasite ‘talks’ the host out of responding by means of a molecular dialog, including contributions of a variety of parasite-derived molecules (PDMs). It must be noted that canine hosts clearly recognize the presence of heartworms, as evidenced by the ample antibody response directed at the parasite, but this response is insufficient to prevent infection or remove the parasite before reproduction occurs. We do not yet have a complete or clear understanding of the components of the immune system that underlie the non-permissive state for parasitic helminths, which may be multi-faceted.

This restricted host range greatly limits the kinds of experiments needed to illuminate crucial aspects of heartworm biology and how the parasite interacts with its host. Previous research on the use of immunocompromised rodents, especially Mongolian jirds (*Meriones unguiculatus*), to host important trichostrongyloid parasites of ruminants [see 10 for review] greatly facilitated screening for new anthelmintics that eliminate these parasites. Similarly, it has been known for some time that immunocompromised mice can host species of filarial parasites for which they are normally non-permissive, including *Brugia malayi* in Severe Combined Immunodeficient (*scid*) mice [11] and adult *Onchocerca volvulus* in transplanted nodules in (*scid*) mice and athymic nude rats [12]. More recently, genetically immunocompromised mouse strains have been reported to serve as permissive hosts for at least part of the life cycle of several filarial species that are important human or veterinary pathogens, including *Onchocerca ochengi*, *O. volvulus* and *Loa loa* [13–15] (Table 1). This work has now been extended to *D. immitis*, as recently described [16–18]. It is now clear that survival and at least partial development of *D. immitis* can be attained in genetically modified mouse strains [16, 18] (Table 1), with the primary short-term goal of facilitating in vivo drug testing, with great utility as a filter to prioritize compounds for testing in dogs. The other, a patent [17], claims survival and development of heartworms in rats immunosuppressed by dosing with a corticosteroid, similar to the process used in jirds for GIN. Production of microfilariae was reported in infected rats in this model, but their abundance and infectivity for mosquitos (and subsequently mammals) were not disclosed. It is possible that the relatively small size of the anatomy of the predilection site for adult heartworms in rodents precludes or limits their development to full reproductive maturity. If so, it could be valuable to explore larger potential species that might be susceptible to infection if immunocompromised, such as older rats, rabbits or guinea pigs. The availability of a model host that could maintain the life cycle of the parasite in mammals would greatly democratize heartworm research, enabling many new labs to pursue studies in this area.

**Table 1:** Immunocompromised rodent models for filarial parasites

Parasite	Host	Host Phenotype	References
*Brugia malayi*	SCID mouse	T cell and B cell deficient	[11]
*Onchocerca volvulus*	SCID mouse, athymic nude rats	As above; T cell deficient rats	[12]
*Brugia malayi, Onchocerca ochengi*	SCID mouse	As above	[13]
*Onchocerca volvulus*	NOD.Cg-*Prkdc*^*scid*^*Il2rg*^*tm1Wjl*^/SzJ (NSG) mice; NOD-*Rag1*^*tm1Mom*^* IL2rg*^*tm1Wjl*^/SzJ (NRG) mice NSG-humanized mice	Defects in macrophages, dendritic cells, complement, T cells, B cells, NK cells, eosinophils, cytokine signalling	[14]
*Loa loa*	SCID mice, NSG mice, RAG −/− γc −/− mice	As above; T cell, B cell and NK cell deficient	[15]
*Dirofilaria immitis*	NSG mice, NRG mice	As above	[16, 18]
*Dirofilaria immitis*	Rat, steroid treated	Broad immunomodulatory effects, deficient in inflammatory responses	[17]

More research may thus be needed to demonstrate that these two rodent models can be of optimal utility by showing that full maturity of adult heartworms can be achieved. Such a milestone would provide an enormous boost to basic and applied research on heartworms. The cost of maintaining heartworms in dogs is substantial, not to mention the prominent ethical concerns around the use of dogs in research (despite the fact that, to date, research on this widely prevalent and very serious infectious disease cannot be done without the use of companion animals as definitive hosts).

## Molecular dialog of the host-parasite interaction: route to a vaccine?

To generate a mature infection, heartworms must defuse the normally highly effective immune response which acts to limit parasitic nematode survival and development in non-permissive hosts. The underlying concept is that parasitic nematodes release a variety of PDMs in a variety of formats and contexts that serve to subvert the normally highly effective immune response that serve to protect hosts from such pathogens [see 19–22]. These PDMs, including metabolites, proteins and microRNAs (miRNAs), constitute a molecular language that is interpreted by permissive hosts to permit establishment and long-term survival of the parasite; the language is not interpretable by non-permissive hosts, which respond to eliminate the parasite soon after invasion. The basis for host-parasite selectivity lies in the fine-tuning of the molecular language by evolution; host receptors for PDMs are specific to those released from a particular parasite species and do not recognize key PDMs from species for which they are not permissive. Identifying the key PDMs and the host receptors/pathways they target to induce a permissive state will open new avenues for therapeutic control, with the goal of converting the permissive (canid/felid) host to non-permissive status by preventing heartworms from eluding the immune response.

Vaccines for protection against filarial infections have been sought for decades and remain of considerable interest in tropical medicine, particularly for onchocerciasis and heartworm; see [23–25]. Significant protection against infections with *D. immitis* was achieved by using chemically abbreviated infections [26, 27] and infection with irradiated L3 larvae [28], demonstrating that exposure to critical parasite antigens can generate a protective antibody response. These observations motivated an intensive search for candidate vaccine antigens, perhaps most notably by a group led by Robert Grieve at the biotechnology company Heska, leading to the filing of some 45 patents for methods of protection against heartworm disease, covering multiple *D. immitis* antigens; see [25]. Unfortunately, no recombinant heartworm antigen was able to generate complete protection against infection as a vaccine, and interest in a vaccine approach waned as the use of highly efficacious ML preventatives increased. It should be noted that, as ML-resistant heartworm populations became recognized, some interest in vaccines has become apparent, exemplified by a recent patent covering intestinal (‘hidden’) antigens of the parasite as potential targets for a protective antibody response [29] (efficacy not described).

Based on the concept that ML preventatives target larval stages of *D. immitis* and other filariae in the mammalian host by blocking the release of essential immunomodulating PDMs [30–34], a vaccine targeted against essential PDMs (assuming at least some are proteins) [35] could, in theory, elicit efficacy equivalent to the MLs, as the antibody response can develop over the roughly 2-month period of the life cycle in which the L4 stage develops. The difficulty of obtaining L4 stage *D. immitis* larvae has limited our ability to define essential PDMs as vaccine targets, although some PDMs from this parasite have been experimentally identified [36–38]. We also do not know whether L4 stages that develop in culture are biologically equivalent to those that develop in a mammalian host. In this regard, rodent models can provide access to naturally developed L4 larvae for comparative studies with those generated by molting from L3 stages in culture. Finally, it should be noted that we do not know how the host eliminates L4 larval stages in immunized dogs or after exposure to MLs, a major gap in developing rational strategies for vaccine antigen identification. The availability of rodent models should greatly facilitate research to address these questions.

An important aspect of the use of genetically modified mouse strains is that they can be reconstituted with immune cell populations derived from other mammals; this has been demonstrated for *O. volvulus* in ‘humanized’ NOD scid gamma (NSG) and NOD.Cg-Rag1^tm1Mom^Il2rg^tm1Wjl^/SzJ/Arc (NRG) immunocompromised mice that house human immune cells [14] and *D. immitis* in caninized mice [16]. In both models, the parasite developed in the reconstituted ‘permissive’ mouse host at least as well as in mice that have no immune cells. The potential power of this system is demonstrated in work with the model filarial parasite *Litomosoides sigmodontis*, which can infect BALB/c mice but not C57Bl/6. Genetically immunocompromised C57Bl/6 mice (RAG2IL-2Rγ-deficient, lacking T, B and natural killer cells) are permissive for *L. sigmodontis*, but adoptive transfer of CD4^+^ T cells from C57Bl/6 mice previously infected with the parasite (but not from naïve mice) reconstituted a non-permissive status in the immunocompromised mice. Further analysis showed that the effective CD4^+^ cells had a TH17 polarized phenotype [39]. It should be relatively straightforward to determine whether the same cell type (or a different one) underlies the non-permissive state in mice for *D. immitis* through reconstitution experiments with wild-type cells in the NSG/NRG strains. Importantly, testing PDMs against these cells to discover those that block the pathways or functions that underlie parasite rejection could reveal the essential host processes that are targeted and the specific PDMs that enable immune evasion; these could be excellent targets for rational vaccine or small molecule therapeutics.

A ‘caninized’ NSG/NRG mouse strain reconstituted with a dog immune system that is permissive for heartworm infection would clearly be an ideal model in which to test candidate vaccines. It is also possible that the immunosuppressed rat model [17] could also be a very useful model for testing vaccine candidates; the fact that ML preventatives retain full activity in this model suggests that the key targets for essential *D. immitis* PDMs can be exploited to reverse the permissive phenotype.

A new vaccine platform could provide a marked increase in the ability to test candidate antigens in these models: mRNA vaccine technology; see, for example, [40–43] as currently exemplified by the Moderna and Pfizer-BioNTech COVID-19 vaccines. The ability to use mRNA rather than purified antigens cuts months from the time needed for an in vivo test and allows rapid, high-throughput experiments that can almost be done on the entire catalog of proteins secreted/excreted by the parasite. It is also possible to mix multiple mRNAs in a single immunization protocol, further enhancing throughput and opening the possibility that a vaccine could include multiple antigens, potentially increasing efficacy to very high levels in an economical and proven vaccine platform.

## Improved drug screening: small animal models

The use of normally non-permissive rodent models for drug assays with clinically important filariae can be expected to improve the efficiency and productivity of antifilarial discovery and development operations. Current paradigms typically employ a model parasite system to prioritize compounds active in preliminary phenotypic or mechanism-based screens; see [44–48]. The major filarial species targeted for chemotherapy, *O. volvulus* and *Wuchereria bancrofti* for humans and *D. immitis* for companion animals, have highly restricted host specificity and so mandate that only the most promising lead compounds can be evaluated in limited human clinical trials. The ability to perform initial in vivo screens that employ the target parasite in a rodent model will allow to more quickly and economically prioritize promising compounds from an intrinsic efficacy and potency perspective, limiting and simplifying the much more expensive studies needed in the target host for product registration. This potential was first demonstrated for *O. volvulus* microfilariae in mice [49], followed by development of a model that maintains transplanted *Onchocerca* nodules and adult males in SCID mice for evaluation of macrofilaricidal activity [13].

The *D. immitis* rodent models [16–18], although proprietary, are nonetheless very valuable and fill an important gap in the screening cascade for new heartworm preventatives. These assays can be anticipated to make the process of discovery of new heartworm prevention medications more productive, requiring much less compound supply and a much faster read-out. Available data also suggest that valuable information about dose-response relationships can be attained, especially in a rat model, for different classes of actives, greatly simplifying dose-ranging studies in dogs.

## Basic pharmacology: macrocyclic lactones and diethylcarbamazine

Much remains uncertain about how heartworm preventatives work in situ. As noted above, a current hypothesis is that MLs prevent the release of immunomodulatory L3/L4 PDMs, revealing the parasite to the immune system, which is then able to generate a non-permissive state that leads to the abrogation of infection. That MLs are effective in both immunocompromised rodent models [16–18] is therefore a conundrum, suggesting that these drugs may have more complex effects at the host-parasite interface. Alternatively, the data may suggest that the immunomodulation caused by parasites in permissive hosts includes both specific and more general immune response and that the parasite is still able to subvert the more general (not parasite species-specific) responses while the specific responses are absent in the modified rodent. If so, pharmacologically blocking the release of PDMs may unleash the general (innate?) response, eliminating the parasite. In may be instructive to consider the differences in relative activity of MLs in the two models. MLs are considerably more active in the rat model than in the mouse model. IVM is 100% effective at dog-equivalent doses in the rat, and moxidectin (MOX) is more potent and equally efficacious, as is the case in dogs [17]. In contrast, 100% efficacy was difficult to reach in the mouse model and higher, multiple doses of IVM and MOX were required for maximum effect [16, 18]. This difference, from the perspective that the extent to which the various arms of the immune response are crippled in the two models, may reveal more about the components of the immune system that are essential for generating a non-permissive state and which are targets for key PDMs.

A second benefit of the rodent models is that light could be shed on the antifilarial pharmacology of diethylcarbamazine (DEC), the first effective commercial heartworm preventative. DEC had to be administered daily during the transmission (mosquito) season and was proposed to act as a preventative only in L3 stages or possibly at the molt between the L3 and L4 stages, which occurs within a few days after the bite of an infected mosquito. As for the MLs, it is thought that this drug reveals the parasite for immune attack [3], and immune-mediated elimination of larvae at this stage prevents development of the parasite to the pathogenic adult stage. A pharmacological explanation for this stage specificity is completely lacking; DEC can be toxic in heartworm-infected dogs because it can have effects on microfilariae and adults [4], as is the case for other filariid species [3]. This poses some interesting and potentially important questions for new therapeutic approaches; it would be highly valuable to discover why DEC does not kill L4 larvae but acts against other mammalian stages of *D. immitis*.

New insight into the receptor that may mediate the effects of DEC on other filarial species has recently been reported [50–52]. DEC is a ligand for filarial Transient Receptor Protein (TRP) channels; these cation channels respond to a variety of stimuli in various settings. Knockdown of the expression of a set of TRP channels in *B. malayi* by RNAi interference limited the activity of DEC on the neuromuscular system of adult parasites. Interestingly, the action of DEC potentiated the effects of emodepside on adult parasites [51], suggesting a possible combination chemotherapy strategy that could be experimentally employed in the new rodent models.

It would be beneficial to extend these observations to *D. immitis* with special attention to all mammalian life cycle stages of the parasite; can differential expression of these channels explain the stage-selective action of DEC in heartworms? Could an L4-specific TRP channel serve as a target for a novel, safe preventative? The recent demonstration of methods for analyzing electrophysiological responses of adult heartworms could be an important complement for such studies [53].

## Understanding resistance to MLs

Small animal models allow economical dose-response experiments using various MLs against susceptible and resistant strains of heartworms. Although IVM did not achieve 100% efficacy as a preventative in the mouse model [16, 18], data show that an IVM-resistant phenotype can be detected. Similar experiments using a single dose of MOX showed that it was both more efficacious than IVM against both a susceptible and a resistant isolate, with a non-significant decrease in efficacy at this dose in the latter [18]. In the immunosuppressed rat model, both IVM and MOX achieved 100% efficacy against a susceptible strain at doses equivalent to those used in dogs, and MOX showed a shift to the right in the dose–response curve against a resistant isolate [17]. More work like this is needed, because the extent of the shift to the right in dose-response curves and their shape in resistant isolates of the parasite can be analyzed to ascertain if all MLs show a similar resistance phenotype, potentially indicating selection of a common mechanism of resistance. Alternatively, if resistant strains differ in the degree of this shift (i.e. in magnitude of resistance) or its shape (slope), it could support the hypothesis that the resistance trait is polygenic or can be attained by more than one mechanism. Such work has rarely been done for heartworms because of the cost and labor involved in performing experiments that require dogs.

Studies done in GIN, particularly *Haemonchus contortus*, suggest that evolution of ML resistance arises in parallel among molecules in this class [54]. However, detection of the phenotype can be complicated by the fact that the magnitude of the use dose over the minimally effective dose may obscure resistance for some members of the class. Typically, further exposure to an ML that remains efficacious against isolates that are resistant to other MLs will eventually select for loss of efficacy for that drug as well [5, 6].

It is not yet clear whether the rodent models can achieve optimal utility: attaining full maturity of adult heartworms, including producing sufficient numbers of microfilariae to propagate the infection, would provide an enormous boost to research on ML resistance in heartworms. Resistant strains could be made increasingly homozygous by repeated cycles of passage in the presence of drug selection followed by targeted breeding in mosquitos, simplifying the quest to identify key genetic changes that underlie the resistance phenotype in comparative. A similar strategy enabled the identification of a relatively small segment of the *H*. *contortus* genome as the site of alleles that cause ML resistance, offering important clues as to the possible cause of the phenotype [55].

Trichostrongyloid nematodes such as *H. contortus* show very high levels of heterozygosity, making identification of resistance-causing alleles a challenge and justifying the inbreeding exercise that led to success in this regard. Susceptible (wild-type) heartworm populations also are heterozygous [56]; resistant strains show increased homozygosity, possibly suggesting a single origin of the resistance phenotype as indicated by population bottlenecking, but considerable efforts in genomic analysis have yet to identify a causative allele(s). Thus, although the presence of two specific single nucleotide polymorphisms (SNPs) is highly correlated with in vivo loss of susceptibility of *D. immitis* microfilariae to MLs [57], the precise cause of this phenotype remains unknown [5, 6]. Enriching resistant parasite strains by repeated rounds of ML treatment and reinfection of a rodent would be expected to be very useful in pinning down the gene(s) and allele(s) responsible for ML resistance in heartworms.

This kind of work is extremely important, as a molecular assay that detects ML-resistance alleles could allow the development and deployment of field assays to survey for the presence of ML-resistant heartworms, preferably in mosquito trap surveys (given the large range of mosquito species that can transmit the infection) [58]. We remain woefully ignorant of the extent of this phenotype and are unable to track its spread.

An equally important factor in the epidemiology of ML-resistant heartworms is determining whether the resistant genotype underlying this phenotype carries with it a fitness deficit. It is likely that the initial selection for ML resistance happened in an area with intensive treatment and high transmission with minimal influx of new mosquitos, so that the large proportion of the total heartworm population this is in refugia (in mosquitos or untreated hosts) could not dilute the resistant individuals that evolved in isolation. If the phenotype incurs a fitness cost, the spread of the resistant population into new areas could be limited. It could be possible, using the rodent models, if they can produce microfilariae, to determine if susceptible heartworms are fitter than their resistant relatives and to quantify the extent of the difference.

## Transgenesis protocols

As noted above, extension of the remarkable functional genomics toolkit available for *C. elegans* to parasitic nematode species, including filariae, has proven to be challenging. A broadly useful method for silencing the expression of target genes to interrogate their function is RNAi, which was pioneered in *C. elegans*. Exposure of a cell or organism to short double-stranded RNAs that specifically recognize the gene of interest can cause significant, though transient, reductions in the mRNA transcribed from that gene, resulting in reduction of the levels of the encoded protein. Adapting this platform to parasitic nematodes, including filariae, has proved to be challenging, cf. [59]. However, it is encouraging that RNAi-mediated knockdown has been reported for filariids, initially in *B. malayi* [60] and subsequently in larval and/or adult stages of other species, cf. [59, 61]. Most of these studies focused on acute effects of RNAi on molting or known or putative drug targets and not on effects on mRNAs that encode proteins essential for survival and development after infection of a host. A notable exception was the description of RNAi in *B. malayi* L3 stages accomplished by exposure of mosquitos to parasite-specific RNAi constructs after ingestion of microfilariae [62]. Targeting a cathepsin-like cysteine protease greatly inhibited larval development of the larva in mosquitos, preventing development fully infectious L3 stages from migrating to the proboscis. Whether RNAi targeted at genes expressed only in L3 and/or L4 stages could persist long enough in this system to produce viable infectious larvae in which those mRNAs were knocked down for studies on their roles in infection and development in the mammalian rather than the arthropod host has not been reported. It should be a priority to extend this technology to *D. immitis*, and the new rodent models for this parasite would provide a facile system with which to evaluate the consequences of gene knockdown on infection.

RNAi-induced phenotypes are temporary and so are limited in what can be accomplished. Developing the ability to generate stably transfected lines of filarial nematodes is an urgent goal, but remains challenging. Altering the genome of *C. elegans* to insert foreign genes, delete endogenous genes or create new alleles of existing genes is readily attainable, but is rarely done in parasitic species; see [59] for review. Transformation of embryos and adult females of *B. malayi* via injection or particle bombardment was reported in 2002 [63], but these methods damaged the parasites and were not compatible with infection studies. It was subsequently reported that exposure of developing *B. malayi* larvae to plasmids in the peritoneal cavity of jirds generated transformed parasites that were viable and generated a subsequent generation that still expressed the transgene [64], although the overall efficiency of transfection was low. Whether the plasmid DNA was incorporated into the parasite genome was not reported, so the stability of the transformation remains uncertain and further work with this protocol does not seem to have been reported.

More recent work reported transfection of L3-L4 *B. malayi* larvae during in vitro culture in the presence of a modified *piggyBac* plasmid and Lipofectamine [65]; transformed larvae developed into adult parasites in culture and were reported to produce microfilariae that expressed a plasmid-encoded reporter gene. Analysis of the genome of transgenic microfilariae revealed that the plasmid had inserted into the targeted area of the parasite genome. In a subsequent work from the same group [66], Liu et al. reported that transformation of *B. malayi* larvae could be accomplished through the CRISPR-Cas9 strategy for inserting foreign DNA into targeted sites in the parasite genome, again producing microfilariae that expressed the transgene in infected jirds. Although the efficiency of the process is not optimal and subsequent work using this protocol has not yet been reported, this has the potential to become a highly valuable platform for functional genomic studies in filariae, especially for the identification and characterization of genes essential for the success of infection.

These experiments demonstrate the exceptional utility of a rodent model for fundamental research. Extending the nascent filarial genomics toolkit to *D. immitis* remains an unfulfilled goal, and the appeal of the recently described rodent models for this parasite is obvious, in conjunction with the pharmacological experiments described above. If the efficacy of MLs in the immunosuppressed rodent models is due to inhibition of the release of PDMs that target remnants of the immune system, L3-L4 heartworm larvae exposed to RNAi construct that target candidate PDMs could identify the key molecules involved in at least some aspects of the parasite-induced immunomodulation that is essential for a successful infection. If the rodent models can be optimized to produce a full life cycle of *D. immitis*, the ability to generate transgenic lines would open many new avenues of research.

## Challenges for heartworm research

Undeniable challenges face the goal of advancing heartworm research with these new paradigms. Perhaps foremost is the lack of sufficient funding for basic research on veterinary parasites in general and companion parasites in particular. Some funding can be secured for research on important livestock parasites and parasites with significant zoonotic potential, but despite the overwhelming contribution of companion animals to the health of their human caretakers, few governmental agencies prioritize research in this area. Funding can often be found for research on companion animals with direct connections to important human diseases, but parasites such as *D. immitis* find commonality with human diseases only in a global perspective, and so struggle to attract support from Western governments. While animal health companies are very interested in heartworms, most external funding is devoted to more clinically relevant research, and budgets for external basic research are generally low. The American Heartworm Society and the National Center for Veterinary Parasitology are important sources of support, but the amounts typically available may not be sufficient to support the level of work that may be needed to make progress. Given concerns about ML resistance, the community of heartworm scientists, including clinicians, academicians and industrial researchers, should work together to develop a strategy for increasing the availability of funds to support novel approaches to this parasite and its control.

A second challenge is the proprietary status of the new rodent models and of mRNA vaccine platforms. One hopes for the holder of the intellectual property to agree to generous policies of permission or licensing terms for the use of these models for research in academic settings.

Despite these challenges, it seems that change is in the air. We are poised to enter a new era of heartworm research that can reveal much about the biology of this parasite and how it interacts with its host on an intimate level. In turn, this knowledge can be expected to lead to new strategies for control that will benefit companion animals and those who care for them.

## Data Availability

All materials and data are present in the manuscript.
